# Modified inverse propensity weighting method to alleviate estimation errors in the model with multiple endogenous variables

**DOI:** 10.1016/j.mex.2023.102513

**Published:** 2023-12-20

**Authors:** Bhubaneswor Dhakal, Geraldine. F.H. McLeod, Andrea Insch, Joseph.M. Boden

**Affiliations:** a Department of Psychological Medicine, University of Otago, PO Box 4345, Christchurch 8140, New Zealand; b Department of Marketing, University of Otago, Dunedin, New Zealand

**Keywords:** Anxiety-disorder, Covariates-exceed-sample-size, Double-confounders, Methods-addressing-endogeneity-problem, Major-depression, Methodological-study, Panel-data, Modified inverse propensity score weighting

## Abstract

Multiple mental health disorders affect on decisions of people. The disorders are also outcomes of other factors. Health studies commonly follow an inverse propensity weight (IPW) method to address estimation errors associated with the presence of one confounder or covariate number exceeding the recommended sample size. However, approaches of IPW appropriate to alleviate the estimation error associated with multiple confounders distributed unequally in the study samples were not explained in our search literature. This study used longitudinal cohort data from Christchurch Health and Development Study and demonstrated IPW approach to address two confounders with similar natures in terms of etiological process. In our sample, some individuals had no mental health disorder at all, while others had either one of depression or anxiety or both. The methodological step to evaluate a new IPW approach include

* Estimated IPWs from all possible combinations of the major depression and anxiety disorder: (a) IPW based on anxiety factor only assuming both mental health problems resulted from the same etiological processes; (b) IPW based on major depression factor only assuming both mental health problems resulted from the same etiological processes; (c) IPW assuming three (independent) categories of etiological processes: neither; either; both of major depression or anxiety disorder, (d) IPW assuming four (independent) categories of etiological processes: neither; major depression only; any anxiety disorder only; both. (e) No IPW or control model (no confounding problem.

• Estimated outcome model with one each IPW at a time and one without IPw (control model).
• Compared fit statistics of all estimated models.
• The IPW derived assuming four categories of etiological processes produced the robust based fit statistics criteria. The study showed significant effects of both mental health problems on investment but the anxiety revealed a stronger effect than that of major depression.

## Graphical abstract

**Figure:**
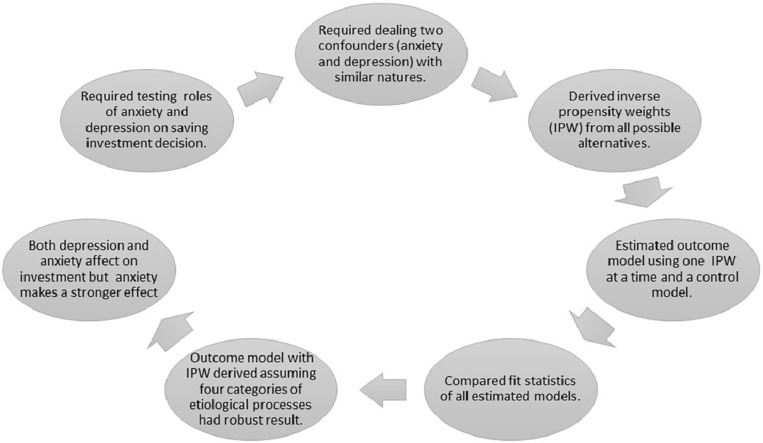

Specifications table

**Table:** 

Subject area:	Health and other social sciences
More specific subject area:	Multiple mental health
Name of your method:	Modified inverse propensity score weighting
Name and reference of original method:	-Li, F., & Li, F. (2019). Propensity score weighting for causal inference with multiple treatments. *The Annals of Applied Statistics, 13*(4), 2389-2415, 2327. -McCaffrey, D. F., Griffin, B. A., Almirall, D., Slaughter, M. E., Ramchand, R., & Burgette, L. F. (2013). A tutorial on propensity score estimation for multiple treatments using generalized boosted models. *Statistics in Medicine, 32*(19), 3388-3414. - Elze, M. C., Gregson, J., Baber, U., Williamson, E., Sartori, S., Mehran, R., ... & Pocock, S. J. (2017). Comparison of propensity score methods and covariate adjustment: evaluation in 4 cardiovascular studies. *Journal of the American College of Cardiology, 69*(3), 345-357.
Resource availability:	N.A

## Method details

Confounding is a commonly reported methodological problem in health research [1] and infers that the effect of the predictors of outcome model may not be exogenous or independent. If the effects of background factors are not controlled, obtained estimates of outcome models may be biased [[1], [2], [3], [4]]. Such problems arise in clinical experimental studies when the number of covariates of interest exceed recommended sample size [5]. Studies follow special methods to address the endogeneity problem [4]. Common methods to address endogeneity and confounding include inverse propensity weighting, g-computation using a system generalized method of momentum (GMM) estimator, the instrumental variable approach, and two-stage estimation methods [[4], [6], [7]]. The choice of methods to investigate any problem depends primarily on the availability of appropriate data, the fit of the available data to apply a particular method, and sources of endogeneity problems (omitted variable problems or simultaneity effects). IPW is the most practiced and convenient approach to address the confounding problems in the health sector [[5], [8]].

When examining multiple mental health problems, the application of many methods is challenging, even for single-variable confounding problems [[4], [6], [7]]. For instance, identification of an appropriate instrumental variable is difficult because an instrumental variable is required to be correlated with the endogenous explanatory variables and have no unmeasured confounding and no direct effect on the outcome (i.e., the instrumental variable may only affect the outcome through the endogenous variable). Variables with such properties rarely exist or may not exist at all [[9], [10]]. Unfortunately, the use of poor-quality instrumental variables may bias the model estimates [11]. Other methods of addressing confounding problems are computationally complex [[4], [6], [7]], particularly if the variables of interest contain multiple subgroups.

One such case is for mental health disorders, in which individuals may experience more than one mental health problem concurrently [[12], [13], [14]]. Some of them especially anxiety and major depression behaves similar etiological processes. Explanatory factors of the problems are overlapping in nature but affect mostly in different magnitudes. Despite the growing application of inverse propensity weighting (IPW) in the field of epidemiology, a review of the literature indicated that the issue of multiple health problems has not previously been considered. A substantial number of studies have examined mental health effects on economic outcomes [[15], [16], [17], [18], [19], [20]], but those studies assumed that the samples have been subject to only one treatment -intervention or exposure -or that the multiple problems (here both depression and anxiety) occur in exclusive groups in the sample as explained by McCaffrey et al. [21]. Methodologically, the application of the IPW approach to address endogeneity problems of similar nature to two variables is not explained and demonstrated.

To address the problem of confounding, these previous studies have used a generalized weight in multiple treatment samples [[18], [21]]. However, this approach does not adequately account for the situations of samples with a mix of mental health problems. Therefore, the power of the conventional IPW approach to control the effect of background factors is questionable in studies of the impact of multiple health problems on savings and investments. However, it is possible to deal with confounding in this situation by employing a modified version of inverse propensity weight (IPW). This is because the IPW approach is assumed to address time-varying confounding and missing data related to the sample selection biases [[22], [23], [24]].

Studies used many propensity weights (e.g., conventional inverse propensity weight, overlapping weight and calibrated weight) approaches to address confounding problems. While each approach has merits and limitations [25], this study focuses on the conventional inverse propensity weight. Against this background, this study aimed to demonstrate advanced approach of estimating inverse propensity weighting to adjust for confounding of anxiety and major depression for an outcome (saving investment in this example).

### The theoretical model

Among many categories (X, Z) of explanatory variables, of the outcome (Y), Z categories of explanatory variables can have a problem of endogeneity. After adjusting the endogeneity effect with the inverse propensity weighting, the estimating equation can be a form of:

$$Y_{\mathrm{ti}}=\partial_{i}\alpha +\partial_{i}\sum_{j}^{J}\beta_{j}X_{\mathrm{ti}}+\partial_{i}\sum_{k}^{K}\beta_{k}Z_{\mathrm{ti}}+\partial_{i}u_{\mathrm{ti}}+{ɛ}_{i}$$

The parameters α, β, u, and ε are: constant, coefficient, time-variant error term, and individual error term respectively, which need to be estimated. Where is ∂_i= weight multiplier of individual sample members calculated at each year and level.

### Data source and participants

This study uses data from the Christchurch Health and Development Study (CHDS) birth cohort. Cohort members were born in mid-1977 (initial n = 1265), participating in 24 waves of assessment at birth, four months, annually from one year to 16 years, and at 18, 21, 25, 30, 35, and 40 years [[12], [26]]. The Health and Disability Ethics Committee (administered by the New Zealand Ministry of Health) provided ethical approval to collect, store and use the data. Trained interviewers administered structured questionnaires face-to-face (or via telephone for overseas cohort members) to collect the data.

This analysis is based on data from cohort members studied at ages 30 years (n=987), 35 years (n=962), and 40 years (n=904) for whom information was available on concurrent mental health and value of savings and investments for at least one assessment from ages 30 to 40 years. These samples represent between 74% and 80.2% of the cohort members surviving to 30 years (n=1231), 35 years (n=1223), and 40 years (n=1221).

### Outcome measure

Value of savings/investments (NZD; 30, 35, and 40 years): At ages 30, 35, and 40 years, cohort members were questioned about whether they had any savings or investments. Savings/investments included money in: savings or trading banks; superannuation schemes; stocks, shares, or debentures; rental properties or other real estate; secured loans; investment or finance companies; building societies or friendly societies; accounts held by lawyers or accountants; or any other investments. Those who had investments were asked for the total realizable value of their investments. Investments reported in currencies other than New Zealand dollars were converted into New Zealand dollars using Purchasing Power Parities for the years 2007 (age 30), 2012 (age 35), and 2017 (age 40) (Organisation for Economic Co-operation and Development (OECD), 2007, 2012, 2017).

### Multiple endogeneity factors

#### Major depression (30-40 years)

At the 30, 35- and 40-year assessments, cohort members were questioned about symptoms of major depression since the previous assessment. The questioning was based on the relevant components of the Composite International Diagnostic Interview (CIDI, WHO, 1993) and DSM-IV criteria (APA, 1994). Using this information, a dichotomous measure was constructed to reflect whether the cohort member met diagnostic criteria for a diagnosis of a major depressive episode for the intervals 25-30, 30-35, and 35-40 years.

#### Any anxiety disorder (30-40 years)

At the 30, 35- and 40-year assessments, cohort members were also asked about a range of anxiety disorders (generalized anxiety disorder, panic disorder, agoraphobia, social phobia, and specific phobia) they may have experienced since the previous assessment, using the same method for determining major depression, above. Using this information, a dichotomous measure was constructed to reflect whether the cohort member met diagnostic criteria for a diagnosis of any anxiety disorder for the intervals 25-30, 30-35, and 35-40 years.

### Methodological construction of the endogenous variables

The two dichotomous classification measures of mental health (internalizing) disorders during each of the three assessment periods (25-30 years; 30-35 years; 35-40 years) were used to create additional variables for the present analyses. These included:

Either mental health (internalizing) disorder: cohort members who met the criteria for either major depression or an anxiety disorder during an assessment period were classified as having a mental health (internalizing) disorder for that assessment period.

• The three-level categorical measure of mental health (internalizing) disorder: Cohort members were classified for each assessment period as to whether they met criteria for: (a) neither mental health disorder; (b) either major depression or anxiety disorder; or (c) both major depression and anxiety disorder.
• The four-level categorical measure of mental health (internalizing) disorder: Cohort members were classified for each assessment period as to whether they met criteria for (a) neither mental health disorder; (b) major depression only; (c) anxiety disorder only; or (d) both major depression and anxiety disorder.

Table 1 shows the definitions and dummy coding of the testing models.

**Table 1:** Definitions and dummy coding of the testing models.

Testing model assumption	Weight calculation model basis	Definition
Binary problem: assumed all mental health disorders result from the same etiological processes	Any anxiety disorder	If anxiety MHA =1, otherwise 0
	Major depression	If depression MHD=1 otherwise 0
	Either major depression or any anxiety disorder.	If either any MHAD=1, otherwise 0
There are three (independent) categories of etiological processes	Three categories (neither; either; both)	If no mental health MHM3=0, else if either of any MHM3=1, else if both MHM3=2
There are four (independent) categories of etiological processes	Four categories (neither; major depression only; any anxiety disorder only; both)	If no mental health MHM4=0, else if depression only MHM4=1, else if anxiety only MHM4=2, else if both MHM4=3
Control model	No weight applied	-

*Note.* MHA – mental health problem of any anxiety disorder; MHD – mental health problem of major depression; MHAD – mental health problem of either major depression or any anxiety disorder; MHM3 – three-category mental health problem of neither, either major depression or any anxiety disorder, both major depression and any anxiety disorder; MHM4 – four-category mental health problem of neither, major depression only, any anxiety disorder only, both major depression and any anxiety disorder.

#### Time-variant covariates of the main outcome model

The time-variant covariates in the outcome model were also gathered at the 30, 35- and 40-year assessments. These covariates included substance and mental health problems (Nicotine abuse/dependence, Cannabis or substance abuse/dependence, and Suicidal ideation), economic circumstances (Highest level of educational attainment, Equivalized net weekly household income, (NZD), Self-employment (yes/no), Number of months unemployed, Household debt, NZD), partner relationship measures (Duration of cohabiting partner relationship (months), Intimate partner violence (IPV) victimization and perpetration score, Number of dependent children).

### Time-variant lagged covariates

Time-variant lagged (t-1) covariates, included in the inverse propensity weight analysis were gathered at the 25, 30- and 35-year assessments. These covariates were excluded from the main outcome models [8].

#### Time-invariant childhood background covariates (< 16 years)

Time-invariant childhood background covariates included a range of measures selected from the CHDS database that had the potential to be correlated with the value of savings/investments and mental health status. These measures spanned measures of:

Socioeconomic background including Father's formal educational qualifications; Averaged family income.

Family functioning includes Parental intimate partner violence, Parental history of illicit substance use; Parental history of criminal offending; Number of Childhood parental changes; Father care and overprotection/control; Childhood physical punishment/maltreatment; Childhood sexual abuse.

Child characteristics include Biological sex; Māori/Pasifika ethnicity; Attention problems; Conduct problems; Novelty seeking; Self-esteem.

See the Online Supplement for further information on the measures listed above.

### Analysis methods

Logistic and multinomial approaches were applied to the data to derive the inverse propensity weights. The choice of approach was dictated by the data classification: logistic for binary data and multinomial for polynomial data. These predictors were used to derive inverse propensity weights as follows.

For individuals who have a single mental health disorder (major depression or anxiety disorder), the inverse propensity score of an individual in the binary case is:

${\text{IPW}}_{ib}=1/\pi_{i}$ if the mental health disorder status of the sample individual =1 (Karyotaki et al.) whereas

$=1/(1-\pi_{i}$) if the mental health disorder status of the sample individual =0 (no).

Where p is the probability of mental health disorder estimated without covariates (i.e., this is a fully (all variable) restricted model). This approach is well explained in Chesnaye et al. (2021).

For individuals with multiple mental health disorders (single mental health disorder, and neither mental health disorder):

$$\text{IPW}=1/\pi_{ij-1}$$

where $=\pi_{ij}$ if the mental health disorder of the sample individual with j category and j-1= 1, 2… and J., else

$$\text{IPW}=1/(1-\pi_{ij0})$$

where $\pi_{ij0}$ = if the sample individual has no mental health disorder (j=no/0).

The IPWs were estimated for each individual year. The analysis produced an extreme IPW for only one observation at year 30 for the ‘anxiety’ approach and at year 35 for the ‘either major depression or anxiety disorder’ approach. In these cases, the weights were truncated to the closest normal weight [27]. The weights were converted into the long format and fitted into a multi-level linear mixed model. Stata 17 (StataCorp, 2021) was used to estimate and apply the IPW for the second level. During the preparation of the descriptive analyses, it was found that three variables (Equivalized net weekly household income (NZD), Household debt (NZD), and Intimate partner violence) had standard deviations larger than their means. These variables were log-transformed for data normalization. Model covariates were selected using forward and backward entry methods.

### Model fit indicators

The performances of the inverse propensity weighting models were evaluated using a multi-level mixed linear model because the dependent variable was measured on a continuous scale across multiple years at ages 30, 35, and 40. Since the parameter values are the sum of the first-level and second-level coefficients, the Monte Carlo test for assessing the performance of the multilevel model is unreliable and cumbersome (Matilainen et al., 2019). Therefore, using the restricted log-likelihood method (RELM), conventional fit statistics were used to compare the fit of the model. The RELM removes all the information about the nuisance parameters, in this case, the mean [28], prior to minimizing the log-likelihood function. It optimizes the log-likelihood value with respect to the variance components instead of the mean component to obtain an unbiased variance estimator. The statistics of pseudo-log-likelihood, AIC, and BIC are used to select robust models from the various alternatives. In addition, the BIC and AIC statistics estimated by REML are not valid for comparing performance between models if the number of degrees of freedom or number of the parameters varied between them [28]. In our case, these do not vary between the models; only the IPW varied across the different models. Therefore, the Log pseudo-likelihood, AIC, and BIC statistics were valid to determine the best approach to estimating inverse propensity weighting. In the analysis, other factors identifying parsimonious results were also considered (Yates et al., 2021), including whether the signs of the statistically significant coefficients were consistent with expectations.

### Comparison of model performance

The model fit statistics (Log pseudo-likelihood, AIC, and BIC) for determining the most robust model (see Methods) are presented in Table 2. The control model was estimated without using any weighting. Inspection of the table's fit statistics profile showed that all other models were superior to the control model. In addition, the four-category model (0 = no major depression or any anxiety disorder; 1 = major depression only; 2 = any anxiety disorder only; 3 = both major depression or any anxiety) had the best-fit profile based on the fit statistics. The results indicated that the weighting approach is statistically the most preferable because the model with the lowest BIC is theoretically better [29].

**Table 2:** Fit Statistics model fit statistics (Log pseudo-likelihood, AIC, and BIC) comparing the various models.

Inverse propensity weighting model	Log pseudo-likelihood	AIC	BIC
Any anxiety disorder only model	-6972.0	13976.1	14069.8
Major depression only model	-6954.8	13941.7	14035.4
Either major depression or any anxiety disorder model	-6992.2	14016.5	14110.2
Three-category model	-6961.9	13955.8	14049.6
Four-category model	-6735.9	13501.8	13589.2
Control model (no weighting correction)	-7380.2	14790.4	14879.1

*Note.* Three category mental health disorder model consisted of neither disorder, either major depression or any anxiety disorder, both major depression and any anxiety disorder; Four category mental health disorder model consisted of neither disorder, major depression only, any anxiety disorder only, both major depression and any anxiety disorder

Table 3 presents the parameter estimates of major depression and anxiety disorder for models using different IPW approaches. Prior examination of the covariates showed that the standard deviations were greater than the mean values for two variables: Equivalized net weekly household income (NZD), Household debt (NZD), and Intimate partner violence. Therefore, these three covariates were log-transformed for data normalization. Detailed results of the models are provided in the Online Supplement Table 2. Inspection of Table 3 shows that the signs of the coefficients in all the models were as expected. The negative signs indicate that both major depression and anxiety problems have an inverse relationship with investment behavior. The coefficients of both anxiety and major depression are statistically significant p<0.05.

**Table 3:** Parameters and statistical significance of major depression and any anxiety disorder, using differing methods of inverse propensity weighting estimation.

Weight model based on	Variable	Co-efficient	Std error	z value	Sig prob	Min 95%	Max 95%
Control (no weighting)	Depression	-0.652	0.223	-2.920	0.003	-1.090	-0.215
	Anxiety	-1.020	0.253	-4.030	0.000	-1.517	-0.523
Any anxiety disorder only	Depression	-0.605	0.261	-2.32	0.020	-1.116	-0.094
	Anxiety	-0.811	0.309	-2.62	0.009	-1.417	-0.204
Major depression only	Depression	-0.535	0.250	-2.14	0.032	-1.026	-0.045
	Anxiety	-1.015	0.315	-3.22	0.001	-1.633	-0.397
Either major depression or any anxiety disorder	Depression	-0.492	0.279	-1.77	0.078	-1.038	0.054
	Anxiety	-0.934	0.332	-2.81	0.005	-1.584	-0.283
Three-category model	Depression	-0.645	0.258	-2.50	0.013	-1.151	-0.139
	Anxiety	-0.876	0.316	-2.77	0.006	-1.496	-0.256
Four-category model	Depression	-0.646	0.317	-2.04	0.041	-1.266	-0.025
	Anxiety	-0.776	0.314	-2.47	0.013	-1.392	-0.161

*Note.* The three-category mental health disorder model consisted of neither disorder, either major depression or any anxiety disorder, both major depression and any anxiety disorder; The four- category mental health disorder model consisted of neither disorder, major depression only, any anxiety disorder only, both major depression and any anxiety disorder. The covariates: Equivalized net weekly household income (NZD,000), Household debt (NZD, 000), Intimate partner violence physical perpetration and victimization score had standard deviations greater than the means, so these measures were log-transformed for data normalization.

## Summary of method

This study used longitudinal data of a birth cohort and assessed the better approach for estimating the IPW to adjust for confounding among cohort members reporting more than one concurrent mental health disorder. The study sample consisted individuals with a range of mental health conditions (no mental health disorder, depression only, anxiety only, and both depression and anxiety). Based on the comparative fit statistics, the IPW derived from most conceptually detailed four-category approach produces the best and most parsimonious model . Previous IPW based studies had not controlled effects of such multiple confounders [21].Our approaches statistically account for the confounding effect of multiple endogenous variables or confounders such as depression and anxiety disorders.

## CRediT author statement

B.D conceptualized the study problem and carried data analysis and drafted the method and result sections. G.M. guided on modality of study and manuscript structuring, provided detail description of data and carried fundamental editing of the manuscript. A.I guided on modality of study and edited the manuscripts in details. J.B. managed fund to study, guided on modality of data analysis and manuscript, and tuned the language of the manuscripts.

## Date of study

This study used the birth cohort data since 1977 and did study with 40 years data in 2023.

## Funding

This work was supported by the 10.13039/501100001505Health Research Council of New Zealand [Programme Grant 16/600].

## Consents

The data were collected with consent from the New Zealand Human Ethics Committee and Health Research Council. This study was not based on clinical trials. The data was collected from cohort members born in 1977. They had signed consent to use the data. This study did not use other sources of materials except citing publicly available references.

## Declaration of Competing Interest

The authors declare that they have no known competing financial interests or personal relationships that could have appeared to influence the work reported in this paper.

## Data Availability

The data associated with this paper is available upon request to the study Director Professor Joseph Boden, email: joseph.boden@otago.ac.nz.

## Appendix Supplementary materials

Image, application 1
