# Supplementary material for: Modified inverse propensity weighting method to alleviate estimation errors in the model with multiple endogenous variables
Source: MethodsX. 2023 Dec 20;12:102513. doi: 10.1016/j.mex.2023.102513 (PMC10772818; doi:10.1016/j.mex.2023.102513)
Supplement: Supplementary file 1 [file mmc1.docx]

**Online supplement for:**

**Modified inverse propensity weighting method to alleviate estimation errors of the model with multiple endogenous variables.**

Bhubaneswor Dhakal a *, Geraldine. F. H. McLeod a , Andrea. Insch b , and Joseph. M. Boden a

a Department of Psychological Medicine, University of Otago, Christchurch, New Zealand;

b Department of Marketing, University of Otago, Dunedin, New Zealand

*Corresponding email: bhubaneswordhakal@gmail.com; bhubaneswor.dhakal@otago.ac.nz.

Secondary title:

Impacts of multiple mental health disorders on adult investment decision: Study with modified inverse propensity weighting methods

Submitted to MethodX

Introduction

Over the life course, an individual or household’s optimal financial decision-making is important for concurrent and future economic security and well-being (Arber et al., 2014; Chang, 2020; Rosen & Wu, 2004; Yogo, 2016; Zemtsov & Osipova, 2016). Financial investment strategies to secure well-being are influenced by complex factors including, for example, mortgage expenses, retirement savings goals, and credit card debt. In addition, it is important to account for other factors influencing financial investment strategies such as cognitive ability, educational achievement, psychological issues, risk perception, and financial literacy (Gomes et al., 2021; Knoll, 2010; Nanda & Banerjee, 2021; Virlics, 2013).

Mental disorders are also an important factor affecting financial decision-making and investment behaviors (Bogan & Fertig, 2013, 2018; Tadesse & Huang, 2022; Tham et al., 2021). Understanding the role of mental disorders and financial decision-making is essential because mental disorders can impair cognitive performance and alter risk perception and risk-taking behaviors (Bogan & Fertig, 2013, 2018; Smout et al., 2020). Given that investment, decision-making involves understanding and managing financial risk tolerance (Baruah & Parikh, 2018), those who have anxiety disorders, characterized by heightened sensitivity to risk and potential danger, may perceive investment as being riskier and therefore may be less willing to invest their money thereby potentially limiting overall financial success (Bogan & Fertig, 2013; Gambetti & Giusberti, 2012; Sun & Shek, 2012; Virlics, 2013). Further, it could be conjectured that depressive disorders characterized by a persistent and/or severely low mood could hamper individuals’ motivation to save or invest for future needs (Hagen, 2011; Knoll, 2010; Smout et al., 2020).

Given the lengthening of human life spans, more resources will be required to sustain longer retirements (Dicker et al., 2018; Ettman et al., 2022). However, evidence is accumulating that mental disorder is increasingly prevalent among younger and college-age individuals (Karyotaki et al., 2020; Twenge et al., 2010), and across society in all age groups (GBD Mental Disorders Collaborators, 2022), thereby decreasing the ability to create adequate future wealth due to the additional economic burden mental disorders place on individuals and their families. Further, while studies may show decreases in rates of mental disorders in older age (Canuto et al., 2018; GBD Mental Disorders Collaborators, 2022), this phenomenon is at odds with the ability to begin a useful savings and investment portfolio past the age of retirement.

A further complication stems from the overall financial burden of mental health problems. These include an individual’s costs of mental health treatment expenses, care costs for the family, and income loss from work disruption (Frijters et al., 2014; Johnston et al., 2019). Employees may have reduced productivity and for their employers, businesses may have additional human resources and employment costs (Bogan & Fertig, 2013; Chisholm et al., 2016; Johnston et al., 2019). Further costs include public costs related to hospital service expenses, GDP loss, and forgone future economic growth (WHO, 2009). These may impact the functioning of the national financial system and the political economy (Gomes et al., 2021; Greenberg et al., 2021) estimated that the incremental economic burden of US adults with major depressive disorder (MDD) was estimated at $US210.5 billion in 2010 (based on 2012 values). However, treatment for mental disorders results in important economic gains which can outweigh the cost of treatment (Chisholm et al., 2016; GBD Mental Disorders Collaborators, 2022; McDaid, 2011; Virlics, 2013). For example, Chisholm et al (2016), estimated the economic benefit of treatment for depression and anxiety was likely to be 2.3 to 3 times higher than the treatment costs.

Taken together, it is important to acknowledge the impact of mental disorders on financial well-being. A number of studies have examined non-economic factors (Knoll, 2010) (such as mental disorders) on financial decision-making and investment behaviors (Alzuabi et al., 2022; Bogan & Fertig, 2013, 2018; Ettman et al., 2022; Ettman et al., 2021; Knoll, 2010; Tadesse & Huang, 2022; Tham et al., 2021). Broadly, these studies have confirmed that mental health problems have a deleterious association with financial well-being. For example, Bogan and Fertig (2018), found a significant negative effect of mental distress on the extent of retirement savings. Alzuabi et al. (2022) also found a negative effect of mental health on household savings and investments.

A large number of studies have investigated the association of wealth with depression (Ettman et al., 2022). While the reverse association is also possible, only a few studies have investigated the impacts of depression and anxiety on savings and investments. Potentially, some studies investigating this issue may not have been published due to non-significant results; although this may have been caused by methodological problems in the analysis (Li & Li, 2019; Roodman, 2009). These issues include: many studies only examining the effect of single mental health problems rather than multiple mental health problems; and among those studies that do examine multiple mental health problems, how confounding is addressed (Li & Li, 2019; Roodman, 2009).

Confounding is a commonly reported methodological issue in health research (Greenland & Morgenstern, 2001) and infers that the effect of the predictors may not be exogenous or independent. If associations are not controlled for the effects of background factors, obtained estimates may be biased (Desai & Franklin, 2019; Ebbes et al., 2021; Greenland & Morgenstern, 2001; Hill et al., 2021). However, it is possible that current methods for estimating the effects may not adequately address the endogeneity problem (Hill et al., 2021).

Common methods to address endogeneity and confounding include inverse propensity weighting, g-computation using a system generalized method of momentum (GMM) estimator, the instrumental variable approach, and two-stage estimation methods (Chatton et al., 2022; Hill et al., 2021; Hogan & Lancaster, 2004). The choice of methods to investigate any problem depends primarily on the availability of appropriate data, the fit of the available data to apply a particular method, and sources of endogeneity problems (omitted variable problems or simultaneity effects). Preferences of the method also depend on common practices in the discipline of the study.

When examining multiple mental health problems, the application of many methods is challenging, even for single-variable confounding problems (Chatton et al., 2022; Hill et al., 2021; Hogan & Lancaster, 2004). For instance, identification of an appropriate instrumental variable is difficult because an instrumental variable is required to be correlated with the endogenous explanatory variables and have no unmeasured confounding and no direct effect on the outcome (i.e., the instrumental variable may only affect the outcome through the endogenous variable). Variables with such properties rarely exist or may not exist at all (Behrman, 2010; Stel et al., 2013). Unfortunately, the use of poor-quality instrumental variables may bias the model estimates (Martens et al., 2006). Other methods of addressing confounding problems are computationally complex (Chatton et al., 2022; Hill et al., 2021; Hogan & Lancaster, 2004), particularly if the variables of interest contain multiple subgroups.

One such case is for mental health disorders, in which individuals may experience more than one mental health problem concurrently (Buchanan et al., 2023; Fergusson & Horwood, 2001; McLeod et al., 2016). Despite the growing application of inverse propensity weighting (IPW) in the field of epidemiology, a review of the literature indicated that the issue of multiple health problems has not previously been considered. A substantial number of studies have examined mental health effects on economic outcomes (Brunie et al., 2014; Frijters et al., 2014; Gili et al., 2013; Li & Li, 2019; Tham et al., 2021; Yu et al., 2021), but those studies assumed that the samples have been subject to only one treatment -intervention or exposure -or that the multiple problems (here both depression and anxiety) occur in exclusive groups in the sample as explained by McCaffrey et al. (2013). Methodologically, the application of the IPW approach to address endogeneity problems of similar nature to two variables is not explained and demonstrated.

To address the problem of confounding, these previous studies have used a generalized weight in multiple treatment samples (Li & Li, 2019; McCaffrey et al., 2013). However, this approach does not adequately account for the situations of samples with a mix of mental health problems. Therefore, the power of the conventional IPW approach to control the effect of background factors is questionable in studies of the impact of multiple health problems on savings and investments. However, it is possible to deal with confounding in this situation by employing a modified version of inverse propensity weight (IPW). This is because the IPW approach is assumed to address time-varying confounding and missing data related to the sample selection biases (Duan et al., 2020; Kim & Steiner, 2016; Seaman et al., 2012).

The literature has demonstrated many approaches to using propensity weights (e.g., conventional inverse propensity weight, overlapping weight, calibrated weight) to address confounding problems. While each approach has merits and limitations (Fuentes et al., 2021), this study focuses on the conventional inverse propensity weight (Ali et al., 2019). Gaining more insight into this under-investigated but important research area will help provide a more accurate estimate of the impact of mental health problems on investment outcomes so that decision-makers can design policies that will reduce the financial burden of mental health problems on individuals, families, and society.

Against this background, this study examines the associations between multiple mental disorders and investment practices using IPW approaches to address confounding. The aims of the study are to:

1. Find the better approach to estimating inverse propensity weighting to adjust for confounding; and

Determine the magnitude of the effect of multiple mental health disorders on savings/investment decisions.

Methods

**Measures**

*Time invariant childhood background factors (0-16 years)*

Socioeconomic background

Father’s formal educational qualifications (at birth of cohort member). Paternal education was assessed at the time of the cohort members’ birth using a 3-point scale that reflected the father’s highest level of educational attainment. This scale was: father lacked educational qualifications; father had secondary (high school) qualifications; and father had tertiary (college) qualifications.

Averaged family income (0-10 years). At each assessment from when the survey child was aged 1–10 years, estimates of the family’s gross annual income from all sources for the previous 12-month period were obtained. This information was used to derive a measure reflecting the average income level available to the family over the 10-year study period. For the current analysis gross income estimates for each year were first classified into deciles of family income, and the resulting decile levels were averaged over the period to obtain an averaged income decile rank for the family.

Family functioning

Parental intimate partner violence (<16 years). The experience of inter-parental violence during childhood (prior to age 16 years) was assessed via participant self-report at age 18, through a series of eight items derived from the Conflict Tactics Scale (Straus, 1979). The eight items used included: (1) threaten to hit or throw something; (2) push, grab or shove other parent; (3) slap, hit or punch other parent; (4) throw, hit, kick or smash something (in the other parent’s presence); (5) kick the other parent; (6) choke or strangle other parent; (7) threaten other parent with a knife, gun or other weapon; (8) call other parent names or criticize other parent (or put other parent down). An overall measure was created by summing the responses for both father- and mother-initiated violence (α= 0.88).

Parental history of illicit substance use (11 years). At the age 11 assessment, the cohort member’s parents were questioned about any history of illicit substance use. Using this information, a dichotomous variable was constructed representing whether or not a history of illicit substance use was reported.

Parental history of criminal offending (15 years). At the age 15 assessment, the cohort member’s parents were questioned about any history of criminal offending. Using this information, a dichotomous variable was constructed representing whether or not a history of criminal offending was reported.

Number of Childhood parental change (0–16 years). Comprehensive data on the child’s family placement and changes of parents were collected at annual intervals from birth to age 16. To assess the extent of parental change, a measure of the child’s exposure to parental change was constructed by counting the number of changes of parents (0–16 years). Parental change was defined as a parent: leaving the home as the result of separation/divorce/death, entering the home due to reconciliation/re-partnering, fostering, or any other change in the custodial parents.

Father care and overprotection/control (<16 years). At age 16 years, cohort members were questioned using the Parental Bonding Instrument (PBI) (Parker et al., 1979) to assess the quality of parenting by their fathers, experienced over the period of childhood. This 25-item scale assesses two broad domains of parenting: parental care and parental over-protection. The parental care dimension measures variations in parent affection, emotional warmth, empathy and closeness. The over-protection dimension assesses variations in parental control, overprotection, intrusion, excessive contact, infantilization. Scale reliabilities were: paternal care (*α* = 0.91) and paternal over-protection (*α* = 0.87). For the purposes of the present analysis, measures of paternal care and paternal over-protection were averaged respectively to create one score of parental care and over-protection.

Childhood physical punishment/maltreatment (<16 years). Exposure to childhood physical punishment/maltreatment was assessed at 18 and 21 years. Cohort members reported on the extent to which their parents used physical punishment during their childhood (prior to age 16 years). If applicable, separate ratings were made for mother figures and father figures. These ratings were then combined into a single four-point scale of parental physical punishment/maltreatment based on the most severe rating at either the 18- or 21-year interview: (1) parents never used physical punishment (4.5% of the sample); (2) parents seldom used physical punishment (78.0% of the sample); (3) at least one parent regularly used physical punishment (11.2% of the sample);(4) at least one parent used frequent or severe punishment or treated the cohort member in a harsh/abusive manner (6.4% of the sample) (Fergusson & Lynskey, 1997). For the purposes of the current analysis, the measure of childhood physical punishment/maltreatment was dichotomized into two groups: 1 = the cohort member experienced regular or severe physical punishment/maltreatment by parents; 0 = the cohort member reported that they experienced occasional or no physical punishment/maltreatment.

Childhood sexual abuse (<16 years). At ages 18 and 21 years, cohort members were questioned about their exposure to any forms of childhood sexual abuse prior to age 16, including: (a) non-contact episodes involving indecent exposure, public masturbation or unwanted sexual propositions; (b) episodes involving sexual contact in the form of sexual fondling, genital contact or attempts to undress the cohort member; and (c) episodes involving attempted or completed vaginal, oral or anal intercourse [49,50]. Using these reports, cohort members were classified into one of four exposure groups reflecting the most severe form of abuse exposure reported at either 18 or 21 years. This classification was: no childhood sexual abuse (76%), non-contact childhood sexual abuse (5%), contact childhood sexual abuse not involving attempted or completed sexual penetration (8%), and severe childhood sexual abuse involving attempted or completed sexual penetration (11%). For the purposes of the current analysis, the measure childhood sexual abuse was dichotomized into two groups: 1 = the cohort member experienced any childhood sexual abuse; 0 = the cohort member did not report any childhood sexual abuse.

Child characteristics

Biological sex (at birth of cohort member). The sex of the cohort member was recorded at the time of the cohort member’s birth.

Māori/Pasifika ethnicity (at birth of cohort member). Children were classified as being either of European (85.4% of the cohort) or Māori/Pasifika ethnicity (14.6%) based on parental reports of ethnic ancestry obtained at the time of the cohort member’s birth.

Attention problems (7-13 years). At ages 7, 9, 11 and 13 years, teacher reports of child behavior problems were gathered using a measure that combined items from the (Rutter et al., 1970) and (Conners, 1969, 1970) parent and teacher questionnaires (Fergusson et al., 1991). This scale assessed the extent to which the child exhibited problems relating to inattention, poor concentration, short attention span, distractibility, restlessness, impulsivity and hyperactivity (Fergusson et al., 1991).

Conduct problems (7-13 years). At ages 7, 9, 11 and 13 years, teacher reports of child behavior problems were gathered using a measure that combined items from the (Rutter et al., 1970) and (Conners, 1969, 1970) parent and teacher questionnaires. This scale assessed the child’s tendencies to conduct disordered and oppositional behaviors including disobedience and defiance of authority, fits of temper and irritability, aggression or cruelty towards others, destruction of property, lying, stealing and similar behaviors (Fergusson et al., 1991).

Novelty seeking (16 years). At the age 16 assessment, cohort members were assessed on the novelty seeking scale of the Tridimensional Personality Questionnaire (Cloninger, 1987). This 31-item scale provides a measure of the extent to an individual considered themselves to be ``impulsive, exploratory, excitable, disorderly and distractible'' (Cloninger, 1987, p.411). Scale items were summed to produce an overall novelty-seeking measure. The internal consistency of the scale was α=0.76

Self-esteem (15 years). At the age 15 assessment, self-esteem was assessed using the Coopersmith Self Esteem Inventory (Coopersmith, 1981). The inventory contains four scales which measure self-esteem "in the social, academic, family and personal areas of experience". The overall measure of self-esteem was generated by summing of the four subscale scores at each age. The internal consistency of this scale was high (α = 0.87).

*Covariates of the main outcome model*

Economic circumstances

Highest level of educational attainment (30-40 years). At ages 30, 35- and 40-years, cohort members were asked to report on their attainment of educational and vocational qualifications. Using these data, cohort members were classified on a four-point scale reflecting their highest level of academic attainment by age 40 years. This scale was: no formal educational qualifications; high school or basic level tertiary qualification (NZQA level 4 or lower); tertiary qualification below degree level (NZQA level 5/6) and university Bachelor’s degree or higher.

Equivalized net weekly household income, NZD (30-40 years). At ages 30, 35 and 40 years, cohort members were questioned about their current net (after tax) weekly income from all sources and (if applicable) that of their partner. From this information, estimates of total net weekly household income were obtained. Incomes reported in currencies other than New Zealand dollars were converted into New Zealand dollars using Purchasing Power Parities (Organisation for Economic Co-operation and Development (OECD), 2007, 2012, 2017). Incomes were annualized by multiplying the weekly income by 52 weeks. Incomes were also truncated to a maximum of $150,000 to avoid the influence of outliers. These estimated incomes were then equivalized for household size and composition using the method described by Jensen (1988). This method provides a set of weights which are used to adjust for the effects of family size and composition. Using this information, a measure of household income was constructed for each of the interview periods 29-30, 34-35 and 39-40 years.

Self-employment (30-40 years). At ages 30, 35- and 40-years, cohort members were asked to report whether they were self-employed in their current (or most recent) job. Using this information self-employment was classified as a dichotomous variable (1=self-employed; 0=not self-employed) for the intervals 29-30, 34-35 and 39-40 years.

Number of months unemployed (30-40 years). At ages 30, 35, and 40 years cohort members were questioned about any periods in which they had been unemployed and seeking work since the previous assessment and the duration of unemployment in months. Using this information, a measure of the total duration of unemployment, in months, was constructed for each of the interview periods: 25-30 years, 30-35 years and 35-40 years.

Household debt, NZD (30-40 years). At ages 30, 35 and 40 years, cohort members were questioned about whether they had any debt they and (if applicable) debts of their partner on student loans, hire purchases, personal loans/finance company loans, credit cards, fines, repayments to WINZ (Work and Income) or other debts, and the total amount owed. Debt reported in currencies other than New Zealand dollars were converted into New Zealand dollars using Purchasing Power Parities (Organisation for Economic Co-operation and Development (OECD), 2007, 2012, 2017). Using this information, a measure of household debt was constructed for each of the interview periods 29-30, 34-35 and 39-40 years.

Partner relationship measures

Duration of cohabiting partner relationship (months) (30-40 years). At ages 30, 35 and 40 years, cohort members were questioned about whether they had a cohabiting partner and the length of time they had been cohabiting in months. Using this information, a duration of cohabiting partner relationship score was calculated for each of the intervals 29-30, 34-35, and 39-40 years based on a sum of the duration in months.

Intimate partner violence (30-40 years). At the 30, 35- and 40-year assessments, cohort members who reported having been in a romantic relationship lasting one month or longer at any time in the past 12 months when aged 29-30 years (n=878), 34-35 years (n = 835) and 39-40 years (n = 800) were questioned about their victimization by and perpetration of intimate partner conflict and violence during the past 12 months. Intimate partner violence was assessed using questionnaires based on the Conflict Tactics Scale version 2 (CTS2) (Straus et al., 1996). Each questionnaire comprises 25 items on four subscales: minor psychological violence, severe psychological violence, physical violence and severe physical violence. The internal consistencies of these scales ranged from α=0.74-0.85. Using this information, two scale scores were constructed for each interval 29-30, 34-35 and 39-40 years reflecting the number of IPV victimization by and perpetration incidents in the relationship.

Number of dependent children (30-40 years). At the 30, 35- and 40-year assessments, cohort members were asked how many dependent children they were currently parenting (including biological, step, adoptive or foster children who were living with the respondent).

Mental health problems

Substance use disorders (30-40 years). At ages 30, 35- and 40-years cohort members were interviewed about aspects of their mental health since the previous assessment. The interview utilized components from the Composite International Diagnostic Interview (World Health Organization, 1993) to assess Diagnostic and Statistical Manual of Mental Disorders, 4th edition (DSM-IV) symptom criteria (American Psychiatric Association, 1994) for substance use disorders (nicotine, alcohol, cannabis abuse or dependence). Using this information, cohort members were classified on dichotomous measures reflecting whether they met the diagnostic criteria for nicotine abuse/dependence, alcohol abuse/dependence or cannabis substance abuse/dependence in each of the time intervals 25–30, 30-35, and 35-40 years

Suicide ideation (30-40 years). At the 30, 35- and 40-year assessments cohort members were questioned using custom-written survey items about whether they had ever thought about killing themselves since the previous assessment and the frequency of such thoughts or attempts (Fergusson et al., 2008). Using this information, cohort members were classified on dichotomous measures reflecting whether they met diagnostic criteria for suicidal ideation in each of the intervals: 25–30, 30–35, and 35-40 years.

Number of life events (30-40 years). At the 30, 35- and 40-year assessments, cohort members were administered a life events scale in which asked them to report on whether they had experienced any of a series of life events in the past 12 months (Boden et al., 2014; Henderson et al., 1981; Holmes & Rahe, 1967). These events spanned a series of domains including: relationship difficulties; separation/divorce; serious illness of the cohort member or close family members; death of close family members; employment difficulties; serious financial problems victimization; and other adverse events. Items about unemployment were not included in this measure due to other unemployment measures being included in the statistical analysis. Using this information, a total life event score was calculated for each of the intervals 29-30, 34-35, and 39-40 years based on a count of the number of adverse life events reported for each interval.

*Time variant lag variables*

In the current analysis, lagged (t-1) versions of the adult covariates were created using the age 25, 30 and 35-year datasets for the analyses at ages 30, 35 and 40 years; described above.

**Sample size and sample bias**

The level of sample attrition raises issues of study reliability. Online Supplement Table 1 shows a comparison of the analysis sample with surviving members of the Christchurch Health and Development Study cohort who were not assessed at age 40.

**Online Supplement Table 1.**

*Associations between surviving members of the Christchurch Health and Development Study cohort included in the analysis sample^a^ and those surviving cohort members who were not assessed at 40 years.*

| **Measure** | **Assessed** (N=904) | **Not assessed** (N=317) | Chi-square. P-value^b^ |
| --- | --- | --- | --- |
| % Female cohort member | 47.7 | 55.2 | 0.021 |
| % Child of single parent at birth of cohort member | 6.3 | 11.7 | 0.002 |
| % of mother aged < 25 years at birth of the cohort member | 46.9 | 52.5 | 0.085 |
| % Mother attained no formal education qualifications by birth of cohort member | 49.6 | 55.4 | 0.075 |
| % Family of low socioeconomic status at birth of the cohort member | 24.1 | 33.1 | 0.002 |
| % Mother smoked during pregnancy with cohort member | 32.7 | 39.8 | 0.024 |

Note: ^a^ The analysis sample was based on those cohort members who were assessed at age 40; the not assessed sample were surviving cohort members who were lost to follow-up at that time; ^b^ Associations were tested for statistical significance using the Chi-square test for independence.

**Online Supplement Table 2.**

*Coefficients of the Four Category Weighted Model.*

| Variable | Co-efficient | Std error | z value | Sig prob | Min 95% | Max 95% |
| --- | --- | --- | --- | --- | --- | --- |
| Year 35 | 2.122 | 0.182 | 11.68 | 0.000 | 1.766 | 2.478 |
| Year 40 | 3.329 | 0.209 | 15.95 | 0.000 | 2.920 | 3.737 |
| Major depression (yes/no) | -0.646 | 0.317 | -2.04 | 0.041 | -1.266 | -0.025 |
| Any anxiety disorder (yes/no) | -0.776 | 0.314 | -2.47 | 0.013 | -1.392 | -0.161 |
| Highest level of educational attainment | 0.963 | 0.118 | 8.17 | 0.000 | 0.732 | 1.194 |
| Equivalized net weekly family income (NZD ,000), log | 0.162 | 0.047 | 3.42 | 0.001 | 0.069 | 0.255 |
| Self-employment (yes/no) | 0.473 | 0.218 | 2.17 | 0.030 | 0.045 | 0.901 |
| Number of months unemployed, log | -0.052 | 0.019 | -2.74 | 0.006 | -0.090 | -0.015 |
| Household debt (NZD ,000) | -0.036 | 0.018 | -2.04 | 0.041 | -0.071 | -0.001 |
| Intimate partner violence physical perpetration and victimization score, log | -0.293 | 0.127 | -2.32 | 0.021 | -0.542 | -0.045 |
| Number of dependent children | -0.254 | 0.081 | -3.16 | 0.002 | -0.412 | -0.096 |
| Interaction between major depression and any anxiety disorder | 0.597 | 0.499 | 1.20 | 0.231 | -0.380 | 1.575 |
| Constant | 3.843 | 0.542 | 7.09 | 0.000 | 2.781 | 4.906 |

Note: NZD = New Zealand Dollars; Four category mental health disorder model consisted of neither disorder, major depression only, any anxiety disorder only, both major depression and any anxiety disorder; Baseline year = 30 years. The amount of investment increased over the years, but the family income variable did not fit with quadratic forms. Covariates included in the models: Equivalized net weekly household income (NZD ,000), Household debt (NZD, 000), Intimate partner violence physical perpetration and victimization score had standard deviations greater than the means, so these measures were log-transformed for data normalization.

**Online Supplement Table 3**

*Value of savings/investment (NZD ,000) reported at assessments 30, 35 and 40 years by the time variant confounding covariates (30, 35 and 40 years).*

|  |  | 30 years | |  | 35 years | |  | 40 years | |
| --- | --- | --- | --- | --- | --- | --- | --- | --- | --- |
| Time variant measures^a^ | Status | Mean (SD) | Corr^b^ |  | Mean (SD) | Corr |  | Mean (SD) | Corr |
| **Substance and Mental Health Problems** |  |  |  |  |  |  |  |  |  |
| Nicotine abuse/dependence (30-40 years) | Yes | 23.0 (76.0) |  |  | 44.3(105.4) |  |  | 72.2 (125.1) |  |
|  | No | 77.5 (155.9) |  |  | 109.4 (167.9) |  |  | 172.1 (190.2) |  |
|  | p sig | <0.001 | -0.156* |  | <0.001 | -0.149* |  | <0.001 | -0.184* |
| Cannabis abuse/dependence (30-40 years) | Yes | 23.8 (93.1) |  |  | 53.6 (132.6) |  |  | 25.1 (37.0) |  |
|  | No | 67.6 (145.7) |  |  | 100.7(162.1) |  |  | 162.9(187.2) |  |
|  | p sig | 0.057 | -0.062 |  | 0.042 | -0.057 |  | <0.001 | -0.128* |
| Suicide ideation (30-40 years) | Yes | 26.5 (77.3) |  |  | 61.3 (131.4) |  |  | 74.2 (135.5) |  |
|  | No | 68.9 (147.8) |  |  | 100.4 (162.2) |  |  | 166.6 (188.1) |  |
|  | p sig | <0.001 | -0.078* |  | 0.087 | -0.047 |  | <0.001 | -0.138* |
| **Economic Circumstances** |  |  |  |  |  |  |  |  |  |
| No formal educational qualifications (30-40 years) | Yes | 20.8 (76.1) |  |  | 36.1 (72.2) |  |  | 60.4 (128.7) |  |
|  | No | 71.0 (149.2) |  |  | 105.0 (165.4) |  |  | 167.0 (187.7) |  |
|  | p sig | <0.001 | 0.153* |  | <0.001 | 0.161* |  | <0.001 | 0.186* |
| Lowest quartile weekly equivalized net household income, NZD (30-40 years) | Yes | 14.3 (57.2) |  |  | 45.6 (31.2) |  |  | 63.2(120.1) |  |
|  | No | 83.7 (160.2) |  |  | 118.23(105.5) |  |  | 190.5 (192.9) |  |
|  | p sig | <0.001 | 0.275* |  | <0.001 | 0.322* |  | <0.001 | 0.408* |
| Self-employment (30-40 years) | Yes | 131.4 (196.5) |  |  | 169.6 (218.6) |  |  | 234.3 (210.5) |  |
|  | No | 58.0 (134.8) |  |  | 84.3 (142.5) |  |  | 138.1 (173.1) |  |
|  | p sig | <0.001 | 0.156* |  | <0.001 | 0.199* |  | <0.001 | 0.213* |
| Highest quartile number of months unemployed (30-40 years) | Yes | 48.0 (130.2) |  |  | 56.6 (127.3) |  |  | 115.4 (165.5) |  |
|  | No | 68.8 (146.3) |  |  | 107.77 (166.2) |  |  | 166.4 (188.4) |  |
|  | p sig | 0.082 | -0.067* |  | <0.001 | -0.107* |  | 0.002 | -0.111* |
| Any household debt, NZD (30-40 years) | Yes | 47.6 (106.7) |  |  | 63.8 (109.8) |  |  | 95.6 (128.6) |  |
|  | No | 68.0 (116.0) |  |  | 110.8 (141.5) |  |  | 180.0 (160.1) |  |
|  | p sig | 0.004 | 0.298* |  | <0.001 | -0.054 |  | <0.001 | -0.067* |
| Cohabiting partner relationship (30-40 years) | Yes | 84.6(163.57) |  |  | 155.0 (172.1) |  |  | 181.2 (192.0) |  |
|  | No | 38.2 (104.0) |  |  | 53.6 (100.6) |  |  | 91.4 (147.9) |  |
|  | p sig | <0.001 | 0.095* |  | <0.001 | 0.041 |  | <0.001 | 0.174* |
| Exposed to intimate partner violence physical perpetration and victimization score (30-40) | Yes | 27.3 (84.6) |  |  | 66.4 (142.4) |  |  | 124.7(170.2) |  |
|  | No | 69.6 (148.3) |  |  | 101.5(162.4) |  |  | 161.9(187.1) |  |
|  | p sig | <0.001 | -0.057 |  | 0.051 | -0.034 |  | 0.070 | -0.063 |
| Any dependent children (30-40 years) | Yes | 53.5 (137.3) |  |  | 99.7 (164.2) |  |  | 170.4 (190.5) |  |
|  | No | 72.4 (147.4) |  |  | 97.6 (156.7) |  |  | 126.4 (168.9) |  |
|  | p sig | 0.047 | -0.082* |  | 0.846 | -0.002 |  | 0.001 | 0.067* |

^a^ Note. The measures in this table have been dichotomized for the purposes of data display; for statistical modelling, all measures were used in their natural metric as described in Methods;

Corr^b^.= Correlation coefficient between investment and covariates and * = significant probability = 0.05

p sig = Significance probability of t test for mean difference between unpaired Yes and No sample groups assuming unequal variance; NZD = New Zealand Dollars

**Online Supplement Table 4**

*Value of savings/investment (NZD ,000) reported at assessments 30, 35 and 40 years by the childhood confounding covariates (<16 years).*

| Time invariant measures^a^ | Status | 30 years | |  | 35 years | |  | 40 years | |
| --- | --- | --- | --- | --- | --- | --- | --- | --- | --- |
|  |  | Mean (SD) | Corr^b^ |  | Mean (SD) | Corr |  | Mean (SD) | Corr |
| **Socioeconomic Background** |  |  |  |  |  |  |  |  |  |
| Father attained no formal educational qualifications by birth of cohort member | Yes | 56.1 (134.6) |  |  | 87.8 (154.6) |  |  | 137.5 (179.7) |  |
|  | No | 77.7 (154.8) |  |  | 113.3 (169.5) |  |  | 181.1 (191.0) |  |
|  | p sig | 0.023 | 0.069* |  | 0.017 | 0.076* |  | 0.001 | 0.119* |
| Lowest quartile of averaged family income (0-10 years) | Yes | 45.9 (121.1) |  |  | 84.1 (145.9) |  |  | 130.7(176.6) |  |
|  | No | 72.6 (150.8) |  |  | 104.7(166.3) |  |  | 167.9(188.0) |  |
|  | p sig | 0.007 | 0.134* |  | 0.075 | 0.111* |  | 0.009 | 0.120* |
| **Family Functioning** |  |  |  |  |  |  |  |  |  |
| Exposed to parental intimate partner violence (<16 years) | Yes | 57.1(130.1) |  |  | 97.7(158.0) |  |  | 155.4(184.6) |  |
|  | No | 71.9 (151.9) |  |  | 99.0(162.4) |  |  | 164.7(188.1) |  |
|  | p sig | 0.107 | -0.084* |  | 0.910 | -0.039 |  | 0.463 | -0.086* |
| Parental history illicit substance use (11 years) | Yes | 56.0(135.2) |  |  | 79.6 (140.6) |  |  | 145.2(181.6) |  |
|  | No | 70.2(149.1) |  |  | 105.8(167.4) |  |  | 163.1(187.9) |  |
|  | p sig | 0.181 | -0.038 |  | 0.024 | -0.064 |  | 0.221 | -0.037 |
| Parental history of criminal offending (15 years) | Yes | 47.2(127.0) |  |  | 85.5(165.3) |  |  | 117.0 (172.5) |  |
|  | No | 68.9(147.8) |  |  | 101.0 (160.2) |  |  | 165.7(187.2) |  |
|  | p sig | 0.094 | -0.054 |  | 0.351 | -0.034 |  | 0.008 | -0.092* |
| Three or more changes of parents (<16 years) | Yes | 35.1(109.2) |  |  | 66.2(139.6) |  |  | 135.4(186.2) |  |
|  | No | 72.6(150.1) |  |  | 106.2(164.9) |  |  | 164.1(185.6) |  |
|  | p sig | 0.000 | -0.106* |  | 0.001 | -0.074* |  | 0.075 | -0.091* |
| Lowest quartile father care (16 years) | Yes | 48.6(125.1) |  |  | 87.3(150.1) |  |  | 142.8(119.4) |  |
|  | No | 74.7(153.2) |  |  | 105.3(165.2) |  |  | 170.2(189.1) |  |
|  | p sig | 0.009 | 0.087* |  | 0.125 | 0.028 |  | 0.059 | 0.102* |
| Highest quartile father overprotection/control (16 years) | Yes | 60.9(142.8) |  |  | 96.5(167.2) |  |  | 141.1 (173.0) |  |
|  | No | 68.4(146.5) |  |  | 101.0 (159.1) |  |  | 168.7(189.9) |  |
|  | p sig | 0.511 | -0.037 |  | 0.7365 | -0.007 |  | 0.067 | -0.081* |
| Exposed to regular/severe childhood physical punishment/maltreatment (< 16 years) | Yes | 62.6 (151.7) |  |  | 81.6(148.3) |  |  | 124.1(176.1) |  |
|  | No | 66.4 (142.4) |  |  | 102.4 (163.6) |  |  | 166.0(187.2) |  |
|  | p sig | 0.765 | -0.001 |  | 0.111 | -0.038 |  | 0.009 | -0.065 |
| Exposed to childhood sexual abuse (< 16 years) | Yes | 35.1(97.5) |  |  | 85.6(153.0) |  |  | 103.8(150.7) |  |
|  | No | 71.1(150.2) |  |  | 101.5 (162.8) |  |  | 168.9(190.0) |  |
|  | p sig | 0.000 | -0.078* |  | 0.265 | -0.026 |  | 0.000 | -0.126* |
| **Child Characteristics** |  |  |  |  |  |  |  |  |  |
| Female gender | Yes | 64.7(143.2) |  |  | 100.9 (161.9) |  |  | 161.5 (143.7) |  |
|  | No | 66.7 (145.2) |  |  | 97.7 (160.8) |  |  | 156.3 (139.3) |  |
|  | p sig | 0.822 | -0.007 |  | 0.815 | -0.008 |  | 0.680 | -0.014 |
| Māori/Pasifika ethnicity | Yes | 40.4(118.9) |  |  | 63.1(132.5) |  |  | 110.8 (162.3) |  |
|  | No | 69.8(147.4) |  |  | 104.6(164.7) |  |  | 165.9(188.3) |  |
|  | p sig | 0.011 | -0.071* |  | 0.002 | -0.089* |  | 0.001 | -0.099* |
| Highest quartile attention problems (7-13 years) | Yes | 37.4(120.3) |  |  | 64.8(134.3) |  |  | 89.8(148.8) |  |
|  | No | 72.6(149.1) |  |  | 107.8(166.5) |  |  | 175.8(190.4) |  |
|  | p sig | 0.001 | -0.111* |  | 0.000 | -0.104* |  | 0.000 | -0.176* |
| Highest quartile conduct problems (7-13 years) | Yes | 47.8(134.4) |  |  | 86.1(165.1) |  |  | 119.2 (173.4) |  |
|  | No | 71.4 (147.3) |  |  | 103.9(160.9) |  |  | 171.3 (188.4) |  |
|  | p sig | 0.027 | -0.094* |  | 0.170 | -0.073* |  | 0.000 | -0.154* |
| Highest quartile novelty seeking (16 years) | Yes | 51.6 (132.4) |  |  | 81.5 (140.4) |  |  | 134.6(169.3) |  |
|  | No | 70.4 (148.8) |  |  | 104.9(166.0) |  |  | 169.2(190.5) |  |
|  | p sig | 0.094 | -0.0479 |  | 0.057 | -0.079* |  | 0.022 | -0.130* |
| Lowest quartile self-esteem (15 years) | Yes | 44.0(114.4) |  |  | 84.2 (155.5) |  |  | 133.7 (178.5) |  |
|  | No | 75.7 (156.2) |  |  | 106.5 (164.1) |  |  | 171.2(188.4) |  |
|  | p sig | 0.001 | 0.121* |  | 0.069 | 0.077* |  | 0.009 | 0.150* |

Note. ^a^ The measures in this table have been dichotomized for the purposes of data display; for statistical modelling, all measures were used in their natural metric as described in Methods; NZD = New Zealand Dollars; Corr^b^ = Correlation coefficient between investment and covariates and * = significant probability = 0.05; p sig = Significance probability of t test for mean difference between unpaired Yes and No sample groups assuming unequal variance.

**Online Supplement Table 5.**

Description of the lagged (t-1) time variant measures at assessments at 25, 30, and 35 years

| Time variant measures^a^ | Status | 25 years |  | 30 years |  | 35 years |  |
| --- | --- | --- | --- | --- | --- | --- | --- |
|  |  | Mean (SD) | Corr^b^ | Mean (SD) | Corr^b^ | Mean (SD) | Corr^b^ |
| **Substance and Mental Health Problems** |  |  |  |  |  |  |  |
| Nicotine abuse/dependence | Yes | 31.4 (95.7) |  | 55.5 (123.9) |  | 78.6 (138.4) |  |
|  | No | 75.1 (153.7) |  | 111.4 (169.0) |  | 174.1 (189.9) |  |
|  | p sig | <0.001 | -0.211* | <0.001 | -0.195* | <0.001 | -0.208* |
| Cannabis abuse/dependence | Yes | 34.0 (97.5) |  | 72.7 (150.6) |  | 66.0 (118.6) |  |
|  | No | 69.5 (148.5) |  | 101.7 (62.9) |  | 166.2 (188.3) |  |
|  | p sig | 0.001 | -0.104* | 0.151 | -0.085* | <0.001 | -0.135* |
| Suicide ideation | Yes | 36.4 (114.5) |  | 63.3 (127.5) |  | 49.8 (118.4) |  |
|  | No | 68.9 (146.6) |  | 102.6(164.3) |  | 163.6 (186.9) |  |
|  | p sig | 0.006 | -0.116* | 0.019 | -0.114* | <0.001 | -0.159* |
| Highest quartile number of life events | Yes | 43.7 (126.3) |  | 86.3 (161.0) |  | 155.3 (189.0) |  |
|  | No | 71.2 (147.8) |  | 102.7 (161.2) |  | 159.6 (185.0) |  |
|  | p sig | 0.009 | -0.115* | 0.183 | -0.170* | 0.774 | -0.052 |
| **Economic Circumstances** |  |  |  |  |  |  |  |
| No formal educational qualifications | Yes | 21.8 (85.4) |  | 35.3 (84.7) |  | 55.5 (119.8) |  |
|  | No | 72.5 (150.0) |  | 106.0 (166.0) |  | 168.9 (188.3) |  |
|  | p sig | <0.001 | 0.276* | <0.001 | 0.259* | <0.001 | 0.297* |
| Lowest quartile weekly equivalized net household income, NZD | Yes | 30.0 (91.40) |  | 52.9 (124.8) |  | 82.9 (144.6) |  |
|  | No | 68.2 (156.9) |  | 116.1 (170.4) |  | 182.9(190.7) |  |
|  | p sig | <0.001 | 0.226* | <0.001 | 0.210* | <0.001 | 0.319* |
| Self-employment | Yes | 62.3 (139.1) |  | 168.9 (207.2) |  | 146.3 (177.9) |  |
|  | No | 96.2 (186.2) |  | 91.8(154.4) |  | 222.5(210.7) |  |
|  | p sig | 0.133 | 0.014 | 0.001 | 0.073* | <0.001 | 0.049 |
| Highest quartile number of months unemployed | Yes | 35.9 (109.1) |  | 76.7(154.0) |  | 114.1 (165.0) |  |
|  | No | 73.3(150.9) |  | 103.7(163.3) |  | 168.4(188.8) |  |
|  | p sig | <0.001 | -0.241* | 0.063 | -0.276* | <0.001 | -0.140* |
| Cohabiting partner relationship | Yes | 73.9 (152.4) |  | 110.5 (170.9) |  | 175.6(190.7) |  |
|  | No | 45.3 (119.3) |  | 83.3(146.5) |  | 118.0 (166.7) |  |
|  | p sig | 0.002 | -0.020 | 0.020 | 0.080 | <0.001 | 0.113* |
| Exposed to intimate partner violence victimization | Yes | 61.0 (164.1) |  | 84.7(163.1) |  | 126.9 (173.2) |  |
|  | No | 65.2 (142.0) |  | 100.8 (162.2) |  | 161.0(186.6) |  |
|  | p sig | 0.850 | -0.028 | 0.453 | -0.043 | 0.187 | -0.048 |
| Perpetration of intimate partner violence | Yes | 51.7 (162.9) |  | 89.5(158.1) |  | 125.1 (172.2) |  |
|  | No | 65.5(142.6) |  | 100.3 (162.5) |  | 160.2 (146.4) |  |
|  | p sig | 0.612 | -0.069* | 0.641 | -0.033 | 0.316 | -0.009 |
| Any dependent children | Yes | 49.9(121.1) |  | 78.0(149.3) |  | 153.8 (183.7) |  |
|  | No | 111.0 (167.9) |  | 111.6(167.7) |  | 167.6(189.5) |  |
|  | p sig | <0.001 | -0.287 | 0.002 | -0.171* | 0.291 | -0.072* |

Note. Lagged variables (t-1) were selected from the prior assessments at 25, 30, and 35 years; NZD = New Zealand Dollars; Corr^b^ = Correlation coefficient between the outcome Value of savings and investments and the covariates, where * = significant probability = 0.05; p sig = Associations were tested for statistical significance using a t-test for mean differences between unpaired Yes and No sample groups assuming unequal variance. The correlation coefficients of the investment value are estimated in their natural metric.

**Discussion**

This study used longitudinal birth cohort data to examine the associations between multiple mental disorders and investment decisions. It had two broad aims: assessing the most appropriate approach for estimating the IPW to adjust for confounding among cohort members reporting more than one concurrent mental health disorder and determining the impact of multiple mental health problems on financial decision-making. The results showed that after evaluating the fit statistics of several models, the most conceptually detailed four-category model was the best and most parsimonious model applied to the data. The IPW approach~~es~~ addressed sub-optimal control for confounding that was common in previous research by statistically accounting for the effect of mental health disorders for individuals across a range of possible exposures (no mental health disorder, depression only, anxiety only, or a combination of depression and anxiety). Previous studies of multiple mental health problems had not accounted for such a broad range of exposures (McCaffrey et al., 2013). Therefore, it is clear that the IPW approach used in the current study may have better corrected for confounding (Chesnaye et al., 2022).

In examining the findings, the present study shows that major depression and anxiety disorder have an adverse effect on the investment behavior of individuals, with both disorders being associated with lower levels of savings/investments in adulthood to age 40. The adverse effects of the depression and anxiety problems on saving investment were quantified as 47.6% fewer savings/investments for those with depression while those with anxiety will have 54.0% fewer savings and investments respectively. Further, this study has shown that using a formula developed by van Garderen and Shah (2002), for those with anxiety disorder, the control model overestimates the magnitude of the effect on cohort members’ value of savings/investments by 10%. However, this impact is not seen in major depression. In terms of the take-home message of the study, the IPW approach can address the endogenous problems of two variables provided that the IPW is derived by accounting attributes of both variables at the same time.

These results have shown it is plausible that these mental health disorders may have hampered individuals’ ability to make financial decisions that provide benefits in the future. For example, depression might create a pessimistic view toward obtaining financial gain from investment of current savings whereas anxiety might create greater risk aversion to investing relative to individuals with no such mental health problems. Gambetti and Giusberti (2012) also found a negative association between anxiety problems and investment decision-making. That study estimated that an individual with an anxiety disorder had 17.3% fewer investments. However, it should be noted that Gambetti and Giusberti (2012) used cross-sectional data and did not investigate the impact of depression on the value of savings and investments. Taken together, mental health problems affect an individual’s work and their efficiency which can result in lower income and savings - vital for investment fund generation (Beck et al., 2014; Kessler, 2012). Other studies have been less successful to address the confounding problem. Bogan and Fertig (2018) used lagged anxiety and depression variables, rather than an IPW approach using contemporaneous measures, finding small and statistically non-significant differences in retirement savings between those with and without mental disorders.

Overall, the findings of the current study make an important contribution to the literature on the impact of mental disorders on financial well-being. This paper provides a way of navigating the challenges of addressing confounding, even when the exposure of interest is categorized in a complex way such as multiple mental health problems by demonstrating an approach for calculating propensity weights (Ali et al., 2019; Fuentes et al., 2021). In addition, this study explored the effect of multiple mental health problems on both savings and investments, whereas most studies have explored only retirement savings or household savings (Bogan & Fertig, 2018).

In addition to the novel contribution of this study, the strengths of this study also include the use of prospective longitudinal birth cohort data and a high sample retention rate (74%) from birth in 1977 to the assessment at age 40. The variables of interest, mental health problems, and concurrent savings and investments were collected as repeated measures at three-time points at ages 30, 35, and 40 years. Further, many childhood background factors spanning socioeconomic background, family functioning, and individual characteristics prior to age 16 were also captured. However, it is also important to address some limitations. The data gathered reflect the historical and cultural context of a birth cohort born in Christchurch, New Zealand in 1977, the extent to which these findings may be generalizable to other samples and populations is unclear.

The IPW weights were evaluated on the dependent variable with a continuous scale in measurement. Whether the weight works in the models with a censor, truncated, and binary dependent variable models, is a subject for future study. The proportion of samples with mental health problems was about 17% which is similar to other international studies (Canuto 2017; Mental Disorders Collaborators, 2022; (Karyotaki et al., 2020; Twenge et al., 2010). Future research may also evaluate whether the method performs similarly to other cases which can have a good mix of samples. This study determined the robustness of that approach of IPW on the model fit criteria. The robustness of the IPW method is worth evaluating in the future with other methods.

In conclusion, this study has shown that after considering control for confounding, major depression, and anxiety disorder were significantly associated with lower rates of savings and investments over the period from 25 to 40 years, suggesting that mental health disorders can have a personal economic impact that may not be realized until much later in life. Other studies also found the importance of treating mental health disorders for other life outcomes. (Canuto 2017; Mental Disorders Collaborators, 2022; Chisholm et al., 2016; McDaid, 2011; Virlics, 2013). This finding underlines the importance of prompt and effective treatment for mental health disorders for economic prosperity. Collectively, the findings suggest that IPW methods should be carefully chosen to evaluate the effects of health status, including mental health, on life outcomes, and that the choice of these models will play a critical role in terms of the estimates obtained and the conclusions drawn from the model.

.

**References**

Ali, M. S., Prieto-Alhambra, D., Lopes, L. C., Ramos, D., Bispo, N., Ichihara, M. Y., Pescarini, J. M., Williamson, E., Fiaccone, R. L., & Barreto, M. L. (2019). Propensity score methods in health technology assessment: principles, extended applications, and recent advances. *Frontiers in pharmacology*, 973.

Alzuabi, R., Brown, S., Gray, D., Harris, M. N., & Spencer, C. (2022). Household saving, health, and healthcare utilization in Japan. *Oxford Economic Papers*, *74*(2), 473-497.

American Psychiatric Association. (1994). *Diagnostic and Statistical Manual of Mental Disorders (4th ed.)*. American Psychiatric Association.

Arber, S., Fenn, K., & Meadows, R. (2014). Subjective financial well-being, income and health inequalities in mid and later life in Britain. *Social Science and Medicine*, *100*, 12-20.

Baruah, M., & Parikh, A. K. (2018). Impact of risk tolerance and demographic factors on financial investment decision. *International Journal of Financial Management*, *8*(1), 1-14.

Beck, A., Crain, L. A., Solberg, L. I., Unützer, J., Maciosek, M. V., Whitebird, R. R., & Rossom, R. C. (2014). The effect of depression treatment on work productivity. *Am J Manag Care*, *20*(8), e294-301.

Behrman, J. R. (2010). Investment in education—inputs and incentives (*Handbook of development economics* (Vol. 5, pp. 4883-4975). Elsevier.

Berkowitz, M. K., & Qiu, J. (2006). A further look at household portfolio choice and health status. *Journal of Banking & Finance*, *30*(4), 1201-1217.

Boden, J. M., Fergusson, D. M., & Horwood, L. J. (2014). Associations between exposure to life stress and alcohol use disorder in a longitudinal birth cohort studied to age 30. *Drug and Alcohol Dependence*, *142*, 154-160. <https://doi.org/10.1016/j.drugalcdep.2014.06.010>

Bogan, V. L., & Fertig, A. R. (2013). Portfolio choice and mental health. *Review of Finance*, *17*(3), 955-992.

Bogan, V. L., & Fertig, A. R. (2018). Mental health and retirement savings: Confounding issues with compounding interest. *Health Economics*, *27*(2), 404-425. <https://doi.org/https://doi.org/10.1002/hec.3579>

Brunie, A., Fumagalli, L., Martin, T., Field, S., & Rutherford, D. (2014). Can village savings and loan groups be a potential tool in the malnutrition fight? Mixed method findings from Mozambique. *Children and Youth Services Review*, *47*, 113-120. <https://doi.org/https://doi.org/10.1016/j.childyouth.2014.07.010>

Buchanan, M., Newton-Howes, G., McLeod, G., & Boden, J. (2023). Life course development following childhood adversity: methods and findings from the Christchurch Health and Development Study. *Longitudinal and Life Course Studies*, 1-17.

Canuto, A., Weber, K., Baertschi, M., Andreas, S., Volkert, J., Dehoust, M. C., Sehner, S., Suling, A., Wegscheider, K., & Ausín, B. (2018). Anxiety disorders in old age: psychiatric comorbidities, quality of life, and prevalence according to age, gender, and country. *The American Journal of Geriatric Psychiatry*, *26*(2), 174-185.

Chang, Y.-L. (2020). Does State Unemployment Insurance Modernization Explain the Trajectories of Economic Security Among Working Households? Longitudinal Evidence from the 2008 Survey of Income and Program Participation. *Journal of Family and Economic Issues*, *41*(2), 200-217.

Chatton, A., Borgne, F. L., Leyrat, C., & Foucher, Y. (2022). G-computation and doubly robust standardisation for continuous-time data: A comparison with inverse probability weighting. *Statistical Methods in Medical Research*, *31*(4), 706-718.

Chesnaye, N. C., Stel, V. S., Tripepi, G., Dekker, F. W., Fu, E. L., Zoccali, C., & Jager, K. J. (2022). An introduction to inverse probability of treatment weighting in observational research. . *Clinical Kidney Journal*, *15*(1), 14-20.

Chisholm, D., Sweeny, K., Sheehan, P., Rasmussen, B., Smit, F., Cuijpers, P., & Saxena, S. (2016). Scaling-up treatment of depression and anxiety: a global return on investment analysis. *The Lancet Psychiatry*, *3*(5), 415-424.

Cloninger, C. (1987). The tridimensional personality questionnaire, version IV. *St. Louis, MO: Department of Psychiatry, Washington University School of Medicine*.

Conners, C. K. (1969). A teacher rating scale for use in drug studies with children. *American Journal of Psychiatry*, *126*(6), 884-888.

Conners, C. K. (1970). Symptom patterns in hyperkinetic, neurotic and normal children. *Child Development*, *41*(3), 667-682.

Coopersmith, S. (1981). *SEI - Self Esteem Inventories*. Consulting Psychologists Press.

Desai, R. J., & Franklin, J. M. (2019). Alternative approaches for confounding adjustment in observational studies using weighting based on the propensity score: a primer for practitioners. *BMJ (Clinical Research Ed.)*, *367*.

Dicker, D., Nguyen, G., Abate, D., Abate, K. H., Abay, S. M., Abbafati, C., Abbasi, N., Abbastabar, H., Abd-Allah, F., & Abdela, J. (2018). Global, regional, and national age-sex-specific mortality and life expectancy, 1950–2017: a systematic analysis for the Global Burden of Disease Study 2017. *The Lancet*, *392*(10159), 1684-1735.

Duan, R., Liang, C. J., Shaw, P., Tang, C. Y., & Chen, Y. (2020). Missing at random or not: a semiparametric testing approach. *arXiv preprint arXiv:2003.11181*.

Ebbes, P., Papies, D., & van Heerde, H. J. (2021). Dealing with endogeneity: A nontechnical guide for marketing researchers (*Handbook of market research* (pp. 181-217). Springer.

Ettman, C. K., Adam, G. P., Clark, M. A., Wilson, I. B., Vivier, P. M., & Galea, S. (2022). Wealth and depression: A scoping review. *Brain and Behavior*, *12*(3), e2486.

Ettman, C. K., Cohen, G. H., Vivier, P. M., & Galea, S. (2021). Savings, home ownership, and depression in low-income US adults. *Social Psychiatry and Psychiatric Epidemiology*, *56*(7), 1211-1219. <https://doi.org/10.1007/s00127-020-01973-y>

Fergusson, D. M., & Horwood, L. J. (2001). The Christchurch Health and Development Study: Review of findings on child and adolescent mental health. *Australian and New Zealand Journal of Psychiatry*, *35*(3), 287-296. <https://doi.org/10.1046/j.1440-1614.2001.00902.x>

Fergusson, D. M., & Horwood, L. J. (2013). The Christchurch Health and Development Study. In P. Joyce, G. Nicholls, K. Thomas, & T. Wilkinson (Eds.), *The Christchurch Experience: 40 Years of Research and Teaching* (pp. 79-87). University of Otago.

Fergusson, D. M., Boden, J. M., & Horwood, L. J. (2008). Exposure to childhood sexual and physical abuse and adjustment in early adulthood. *Child Abuse & Neglect*, *32*, 607-619.

Fergusson, D. M., Horwood, L. J., & Lloyd, M. (1991). Confirmatory factor models of attention deficit and conduct disorder. *Journal of Child Psychology and Psychiatry*, *32*(2), 257-274.

Fergusson, D. M., & Lynskey, M. T. (1997). Physical punishment/maltreatment during childhood and adjustment in young adulthood. *Child Abuse & Neglect*, *21*(7), 617-630.

Frijters, P., Johnston, D. W., & Shields, M. A. (2014). The effect of mental health on employment: evidence from Australian panel data. *Health Economics*, *23*(9), 1058-1071.

Fuentes, A., Lüdtke, O., & Robitzsch, A. (2021). Causal inference with multilevel data: A comparison of different propensity score weighting approaches. *Multivariate Behavioral Research*, 1-24.

Gambetti, E., & Giusberti, F. (2012). The effect of anger and anxiety traits on investment decisions. *Journal of Economic Psychology*, *33*(6), 1059-1069.

GBD Mental Disorders Collaborators. (2022). Global, regional, and national burden of 12 mental disorders in 204 countries and territories, 1990–2019: a systematic analysis for the Global Burden of Disease Study 2019. *The Lancet Psychiatry*, *9*(2), 137-150.

Gili, M., Roca, M., Basu, S., McKee, M., & Stuckler, D. (2013). The mental health risks of economic crisis in Spain: evidence from primary care centres, 2006 and 2010. *The European Journal of Public Health*, *23*(1), 103-108.

Gomes, F., Haliassos, M., & Ramadorai, T. (2021). Household finance. *Journal of Economic Literature*, *59*(3), 919-1000. <https://doi.org/DOI>: 10.1257/jel.20201461

Greenberg, P. E., Fournier, A.-A., Sisitsky, T., Simes, M., Berman, R., Koenigsberg, S. H., & Kessler, R. C. (2021). The Economic Burden of Adults with Major Depressive Disorder in the United States (2010 and 2018). *PharmacoEconomics*, *39*(6), 653-665. <https://doi.org/10.1007/s40273-021-01019-4>

Greenland, S., & Morgenstern, H. (2001). Confounding in Health Research. *Annual Review of Public Health*, *22*(1), 189-212. <https://doi.org/10.1146/annurev.publhealth.22.1.189>

Gurka, M. J. (2006). Selecting the best linear mixed model under REML. *The American Statistician*, *60*(1), 19-26.

Hagen, E. H. (2011). Evolutionary theories of depression: a critical review. *The Canadian Journal of Psychiatry*, *56*(12), 716-726.

Henderson, S., Byrne, D. G., & Duncan-Jones, P. (1981). *Neurosis and the social environment* (Vol. 27). Academic Press.

Hill, A. D., Johnson, S. G., Greco, L. M., O’Boyle, E. H., & Walter, S. L. (2021). Endogeneity: A review and agenda for the methodology-practice divide affecting micro and macro research. *Journal of Management*, *47*(1), 105-143.

Hogan, J. W., & Lancaster, T. (2004). Instrumental variables and inverse probability weighting for causal inference from longitudinal observational studies. *Statistical Methods in Medical Research*, *13*(1), 17-48.

Holmes, T. H., & Rahe, R. (1967). The Social Readjustment Rating Scale. *Journal of Psychosomatic Research*, *11*, 213-218.

Jensen, J. (1988). *Income equivalencies and the estimation of family expenditures on children*. (Unpublished). Retrieved 11 November 2014 from <http://archive.stats.govt.nz/browse_for_stats/people_and_communities/Households/energy-hardship-report/appendix-4.aspx>

Johnston, K. M., Powell, L. C., Anderson, I. M., Szabo, S., & Cline, S. (2019). The burden of treatment-resistant depression: a systematic review of the economic and quality of life literature. *Journal of Affective Disorders*, *242*, 195-210.

Karyotaki, E., Cuijpers, P., Albor, Y., Alonso, J., Auerbach, R. P., Bantjes, J., Bruffaerts, R., Ebert, D. D., Hasking, P., & Kiekens, G. (2020). Sources of stress and their associations with mental disorders among college students: results of the world health organization world mental health surveys international college student initiative. *Frontiers in psychology*, *11*, 1759.

Kessler, R. C. (2012). The costs of depression. *Psychiatric Clinics*, *35*(1), 1-14.

Kim, Y., & Steiner, P. (2016). Quasi-Experimental Designs for Causal Inference. *Educ Psychol*, *51*(3-4), 395-405. <https://doi.org/10.1080/00461520.2016.1207177>

Knoll, M. A. (2010). The role of behavioral economics and behavioral decision making in Americans' retirement savings decisions. *Soc. Sec. Bull.*, *70*, 1.

Li, F., & Li, F. (2019). Propensity score weighting for causal inference with multiple treatments. *The Annals of Applied Statistics*, *13*(4), 2389-2415, 2327. <https://doi.org/10.1214/19-AOAS1282>

Martens, E. P., Pestman, W. R., de Boer, A., Belitser, S. V., & Klungel, O. H. (2006). Instrumental variables: application and limitations. *Epidemiology*, 260-267.

McCaffrey, D. F., Griffin, B. A., Almirall, D., Slaughter, M. E., Ramchand, R., & Burgette, L. F. (2013). A tutorial on propensity score estimation for multiple treatments using generalized boosted models. *Statistics in Medicine*, *32*(19), 3388-3414.

McDaid, D. (2011). Making the long-term economic case for investing in mental health to contribute to sustainability. *European Union*.

McLeod, G. F. H., Horwood, L. J., & Fergusson, D. M. (2016). Adolescent depression, adult mental health and psychosocial outcomes at 30 and 35 years. *Psychological Medicine*, *46*(7), 1401-1412. <https://doi.org/10.1017/S0033291715002950>

Morgan, A., Davies, M., & Ziglio, E. (Eds.). (2010). *Health Assets in a Global Context: Theory, Methods, Action*. Springer. <https://doi.org/DOI> 10.1007/978-1-4419-5921-8

Nanda, A. P., & Banerjee, R. (2021). Consumer’s subjective financial well‐being: A systematic review and research agenda. *International Journal of Consumer Studies*, *45*(4), 750-776.

Organisation for Economic Co-operation and Development (OECD). (2007). *Purchasing Power Parities (PPPs) for OECD Countries since 1980*. Retrieved 4 June 2008 from <https://data.oecd.org/conversion/purchasing-power-parities-ppp.htm>

Organisation for Economic Co-operation and Development (OECD). (2012). *Purchasing Power Parities (PPPs) for OECD Countries since 1980*. Retrieved 10 April 2014 from <https://data.oecd.org/conversion/purchasing-power-parities-ppp.htm>

Organisation for Economic Co-operation and Development (OECD). (2017). *Purchasing Power Parities (PPPs) for OECD Countries since 1980*. Retrieved 10 April 2014 from <https://data.oecd.org/conversion/purchasing-power-parities-ppp.htm>

Parker, G., Tupling, H., & Brown, L. B. (1979). A parental bonding instrument. *British Journal of Medical Psychology*, *52*, 1-10.

Petchko, K. (2018). *How to write about economics and public policy*. Academic Press.

Roodman, D. (2009). How to do xtabond2: An introduction to difference and system GMM in Stata. *The stata journal*, *9*(1), 86-136.

Rosen, H. S., & Wu, S. (2004). Portfolio choice and health status. *Journal of Financial Economics*, *72*(3), 457-484.

Rutter, M., Tizard, J., & Whitmore, K. (1970). *Education, Health and Behaviour*. Longmans.

Seaman, S. R., White, I. R., Copas, A. J., & Li, L. (2012). Combining multiple imputation and inverse-probability weighting. *Biometrics*, *68*(1), 129-137. <https://doi.org/10.1111/j.1541-0420.2011.01666.x>

Smout, A., Newton, N. C., Slade, T., O’Donoghue, B., & Chapman, C. (2020). The relationship between early risk-taking behavior and mental health problems among a nationally representative sample of Australian youth. *Journal of Affective Disorders*, *272*, 239-248.

Stel, V. S., Dekker, F. W., Zoccali, C., & Jager, K. J. (2013). Instrumental variable analysis. *Nephrology Dialysis Transplantation*, *28*(7), 1694-1699.

Straus, M. A. (1979). Measuring intrafamily conflict and violence: The conflict tactics (CT) scale. *Journal of Marriage and Family*, *41*, 75-88.

Stürmer, T., Webster-Clark, M., Lund, J. L., Wyss, R., Ellis, A. R., Lunt, M., Rothman, K. J., & Glynn, R. J. (2021). Propensity score weighting and trimming strategies for reducing variance and bias of treatment effect estimates: a simulation study. *American Journal of Epidemiology*, *190*(8), 1659-1670.

Sun, R. C. F., & Shek, D. T. L. (2012). Positive youth development, life satisfaction and problem behaviour among Chinese adolescents in Hong Kong: A replication. *Social Indicators Research*, *105*(3), 541-559. <https://doi.org/10.1007/s11205-011-9786-9>

Tadesse, A., & Huang, J. (2022). Women’s participation in a savings group and depression: A community-based financial capability intervention in Mozambique. *Global Social Welfare*, 1-11.

Temel Nalın, H. (2013). Determinants of household saving and portfolio choice behaviour in Turkey. *Acta Oeconomica*, *63*(3), 309-331.

Tham, W. W., Sojli, E., Bryant, R., & McAleer, M. (2021). Common mental disorders and economic uncertainty: evidence from the COVID-19 pandemic in the US. *PLoS ONE*, *16*(12), e0260726.

Twenge, J. M., Gentile, B., DeWall, C. N., Ma, D., Lacefield, K., & Schurtz, D. R. (2010). Birth cohort increases in psychopathology among young Americans, 1938–2007: A cross-temporal meta-analysis of the MMPI. *Clinical Psychology Review*, *30*(2), 145-154.

van Garderen, K. J., & Shah, C. (2002). Exact interpretation of dummy variables in semilogarithmic equations. *The Econometrics Journal*, *5*(1), 149-159.

Virlics, A. (2013). Investment decision making and risk. *Procedia Economics and Finance*, *6*, 169-177.

Ward, E. J. (2008). A review and comparison of four commonly used Bayesian and maximum likelihood model selection tools. *Ecological Modelling*, *211*(1-2), 1-10.

World Health Organization. (1993). *Composite International Diagnostic Interview (CIDI)*.

Yogo, M. (2016). Portfolio choice in retirement: Health risk and the demand for annuities, housing, and risky assets. *Journal of Monetary Economics*, *80*, 17-34.

Yu, Y., Zhang, M., Shi, X., Caram, M. E., Little, R. J., & Mukherjee, B. (2021). A comparison of parametric propensity score‐based methods for causal inference with multiple treatments and a binary outcome. *Statistics in Medicine*, *40*(7), 1653-1677.

Zemtsov, A. A., & Osipova, T. Y. (2016). Financial wellbeing as a type of human wellbeing: theoretical review. *The European Proceedings of Social & Behavioural Sciences EpSBS*, *7*, 385-392.
